# Research advances and future directions in female ADHD: the lifelong interplay of hormonal fluctuations with mood, cognition, and disease

**DOI:** 10.3389/fgwh.2025.1613628

**Published:** 2025-07-07

**Authors:** J. J. Sandra Kooij, Maxime de Jong, Jessica Agnew-Blais, Silvia Amoretti, Kathrine Bang Madsen, Isabella Barclay, Sven Bölte, Charlotte Borg Skoglund, Thomas Broughton, Sara Carucci, Dorenda K. E. van Dijken, Julia Ernst, Blandine French, Matilda A. Frick, Cédric Galera, Annabeth P. Groenman, Helena Kopp Kallner, Julia Kerner auch Koerner, Sarah Kittel-Schneider, Iris Manor, Joanna Martin, Emilia Matera, Valeria Parlatini, Alexandra Philipsen, Josep Anthoni Ramos-Quiroga, Iris L. Rapoport, Karl Lundin Remnélius, Amandine Sénéquier, Lisa Thorell, Janneke M. E. Wittekoek, Dora Wynchank

**Affiliations:** ^1^ Department of Psychiatry, Amsterdam University Medical Center/VUmc, Amsterdam, Netherlands; ^2^ Expertise Center Adult ADHD, PsyQ, Psycho-medical Programs, The Hague, Netherlands; ^3^ Department of Psychology, School of Biological and Behavioural Sciences, Queen Mary University of London, London, United Kingdom; ^4^ Group of Psychiatry, Mental Health and Addictions, Vall D'Hebron Research Institute (VHIR), Biomedical Research Networking Centre Consortium on Mental Health (CIBERSAM), Barcelona, Spain; ^5^ Department of Clinical Research, University of Southern Denmark, Odense, Denmark; ^6^ National Centre for Register-based Research, Department of Public Health, Aarhus University, Aarhus, Denmark; ^7^ Centre for Neuropsychiatric Genetics and Genomics & Wolfson Centre for Young People’s Mental Health, Cardiff University, Cardiff, United Kingdom; ^8^ Center of Neurodevelopmental Disorders (KIND), Department of Women’s and Children’s Health, Centre for Psychiatry Research, Karolinska Institutet & Region Stockholm, Stockholm, Sweden; ^9^ Child and Adolescent Psychiatry, Stockholm Health Care Services, Region Stockholm, Stockholm, Sweden; ^10^ Curtin Autism Research Group, Curtin School of Allied Health, Curtin University, Perth, WA, Australia; ^11^ Department of Womens and Childrens Health, Uppsala University, Uppsala, Sweden; ^12^ Department of Clinical Neuroscience, Karolinska Institutet, Stockholm, Sweden; ^13^ Department of Medical Sciences and Public Health, University of Cagliari and Child and Adolescent Unit P.O. Microcitemico ASL Cagliari, Cagliari, Italy; ^14^ Department of Gynaecology, OLVG Hospital, Amsterdam, Netherlands; ^15^ Department of Child and Adolescent Psychiatry and Psychotherapy, Central Institute of Mental Health, Medical Faculty Mannheim, Heidelberg University, Mannheim, Germany; ^16^ German Center for Mental Health (DZPG), Partner Site Mannheim-Heidelberg-Ulm, Mannheim, Germany; ^17^ School of Psychology, University of Nottingham, Nottingham, United Kingdom; ^18^ Institute of Mental Health, University of Nottingham, Nottingham, United Kingdom; ^19^ Department of Medical Sciences, Uppsala University, Uppsala, Sweden; ^20^ Department of Psychology, Stockholm University, Stockholm, Sweden; ^21^ University of Bordeaux, INSERM, BPH, U1219, Bordeaux, France; ^22^ Department of Child and Adolescent Psychiatry, Centre Hospitalier Perrens, Bordeaux, France; ^23^ Research Institute of Child Development and Education, University of Amsterdam, Amsterdam, Netherlands; ^24^ Department of Child and Adolescent Psychiatry, University Medical Center Groningen, University of Groningen, Groningen, Netherlands; ^25^ Accare Child Study Center, Groningen, Netherlands; ^26^ Department of Clinical Sciences, Danderyd Hospital Karolinska Institutet, Stockholm, Sweden; ^27^ Department of Obstetrics and Gynecology, Danderyd Hospital, Stockholm, Sweden; ^28^ Institute for Psychology in Education, University of Münster, Münster, Germany; ^29^ IDeA - Center for Research on Individual Development and Adaptive Education of Children at Risk, Frankfurt am Main, Germany; ^30^ Department of Psychiatry and Neurobehavioural Science, University College Cork, Cork, Ireland; ^31^ APC Microbiome, University College Cork, Cork, Ireland; ^32^ ADHD Clinic, Geha Mental Health Center, Petach-Tiqva, Israel; ^33^ Department of Precision and Regenerative Medicine and Ionic Area, University of Bari, Bari, Italy; ^34^ Child and Adolescent Psychiatry, University of Southampton/Hampshire, Southampton, United Kingdom; ^35^ IOW Healthcare NHS Foundation Trust, Southampton, United Kingdom; ^36^ Department of Psychiatry and Psychotherapy, University Hospital Bonn, Bonn, Germany; ^37^ Department of Mental Health, Hospital Universitari Vall d’Hebron, Barcelona, Spain; ^38^ Department of Psychiatry and Forensic Medicine, Universitat Autònoma de Barcelona, Barcelona, Spain; ^39^ Research Institute of Child Development and Education, University of Amsterdam, Amsterdam, Netherlands; ^40^ HeartLife Clinics Utrecht, Utrecht, Netherlands

**Keywords:** ADHD, female, consensus, sex hormones, lifespan, sex specific, menstrual cycle, self-reported needs

## Abstract

**Introduction:**

Attention-Deficit/Hyperactivity Disorder (ADHD) in girls and women is under-recognised and under-researched, despite increasing awareness of clinical challenges and unmet needs. This review by the Eunethydis Special Interest Group on Female ADHD, addresses current knowledge and identifies research gaps for future work. Issues in women with ADHD across the lifespan such as late diagnosis, pubertal development, sexual health, hormonal birth control, executive function difficulties, and gynaecological disorders associated with ADHD are highlighted.

**Methods:**

The review synthesises existing literature and self-reported experiences of women with ADHD to explore the impact of hormonal fluctuations [puberty, menstrual cycle, pregnancy, (peri)menopause] on ADHD symptoms and mood disturbances. It examines the interplay of oestrogen and progesterone with dopaminergic pathways, when periods of lower oestrogen may affect cognition, as well as the manifestation of executive function deficits, and the intersection of ADHD with reproductive health.

**Results:**

Hormonal transitions exacerbate ADHD symptoms and mood disturbances, yet pharmacological research and tailored treatments are lacking. Executive function deficits manifest differently in girls and women with ADHD and are influenced by neuropsychological and neurobiological profiles. Diagnostic practices and sociocultural factors contribute to delayed diagnoses, increasing the risk of comorbidities, impaired functioning, and diminished quality of life. Undiagnosed women have increased vulnerability to premenstrual dysphoric disorder, postpartum depression, and cardiovascular disease during perimenopause.

**Discussion:**

Longitudinal, sex-specific studies incorporating hormonal status and lived experience are needed. Individualised interventions should be developed to address the unique needs of girls and women with ADHD. Addressing these gaps will advance more equitable diagnosis, management, and support for girls and women with ADHD, improving outcomes across the female lifespan.

## 1 Introduction

In October 2024, a “Special Interest Group (SIG) on Female ADHD” was initiated at the Annual Meeting of Eunethydis in Cagliari, Italy. Eunethydis is the European Network for Hyperactivity Disorders, an international network that aims to facilitate high quality science of Attention-Deficit/Hyperactivity Disorder (ADHD), and its wider societal impact through clinical and basic science researchers collaborating across Europe and beyond. Forty-two researchers joined the SIG, and many participated in writing this first paper on “Female ADHD**:** Current Research and Future Directions”. We used the self-reported research needs of women with ADHD as our starting point (detailed below). The aim of this paper is to identify what we know and what we need to know about people assigned female at birth having ADHD, formulating the next research questions and collaborating internationally to speed up the progress.

### 1.1 Self-reported research needs of women with ADHD

Recently, ADDitude, an online media platform that represents the voice of, and advocates for, the ADHD community, inventoried the research needs identified by 750 women with self-reported ADHD (1). These results, similar to those produced by other authors, and by the Duke Center in 2024 (with data from 1,350 women, mostly with self-reported ADHD), highlighted hormonal symptoms and their impact on ADHD symptoms and mood as an area that is both important and needs to be understood better (Table 1) (2).

**Table 1:** Research needs according to ADDitude (1–6) and the Duke center (7–10).

1. Is the onset of puberty associated with worsened ADHD symptoms in women, and if so, why?
2. Why is menstruation in ADHD more commonly associated with debilitating symptoms of Premenstrual Syndrome (PMS) and Premenstrual Dysphoric Disorder (PMDD), and what treatment strategies are successful?
3. While the risk for postpartum depression (PPD) and anxiety among women with ADHD is 5× increased, why are treatment strategies aimed at women with ADHD still lacking?
4. Why do women with ADHD experience exacerbation of ADHD symptoms in perimenopause and how can this be addressed and treated?
5. What hormonal contraceptives are the best for women with ADHD and why?
6. What menopausal hormone therapy is the best for women with ADHD and why?
7. What is the role of hormones in ADHD during episodes of reduced oestrogen?
8. What do we know about cognitive decline in post-menopausal women with ADHD
9. What are the unique harms of a late or incorrect diagnosis in women with ADHD?
10. What are executive function challenges among girls and women with ADHD?

### 1.2 Self-reported symptoms related to hormonal changes across the lifespan

The overwhelming cry for research into “hormonal issues and ADHD” is hardly surprising when one considers the self-reported struggles of women with ADHD throughout life, with exacerbations of symptoms in different periods. According to ADDitude, women with ADHD reported that their adolescence was marked by feelings of sadness or depression (70%), rejection sensitive dysphoria (63%), and greater worry or anxiety (58%). Throughout the reproductive phase of their lives, two-thirds of women said they had experienced PMS and/or PMDD. They experienced the following symptoms in their premenstrual week: irritability (80%), mood swings (79%), cramps or discomfort (79%), tension/anxiety (68%), and lack of focus/concentration (66%).

During pregnancy, for 20% of women, this episode brought greater focus, drive, organisation, and improved sleep. However, 44% of women noticed no difference in ADHD symptoms during pregnancy, perhaps because raised hormone levels were offset by the fact that 98% of women discontinued stimulant medication use. Another 36% said their ADHD symptoms worsened in pregnancy with exhaustion, poor memory, emotional dysregulation, and sensitivity.

The rate of self-reported PPD was 61%. Women reported that symptoms of PPD lasted roughly one year, and included the following: crying spells (76%), feelings of worthlessness, shame, guilt, or inadequacy (72%), mood swings (66%), irritability (62%), lack of concentration (58%), and sleep problems (57%).

In 2022, ADDitude also surveyed 4,000 women with ADHD about the impact of (peri)menopause. 70% of women said ADHD had a “life-altering” impact in their 40s and 50s, with 50% of women calling their ADHD “extremely severe”. The most debilitating symptoms were procrastination and time management (79%), working memory problems (74%), feelings of being overwhelmed (72%), and greater disorganisation (70%).

The conclusion is that there has been a strong call from the community for more research that needs to be taken seriously. Current knowledge however is relatively limited, we therefore aim in this Position paper to provide a comprehensive summary and discuss the available knowledge in the following Subsections:

1. The unique harms of late or incorrect diagnosis
2. The role of hormones in female ADHD
3. The executive function challenges in girls and women with ADHD
4. Pubertal development in girls with ADHD
5. Sexual health and risky sexual behaviour in girls with ADHD
6. The benefits and risks of hormonal birth control for women with ADHD
7. Maternal ADHD in the peripartum period
8. Hormonal and gynaecological disorders associated with ADHD [Polycystic Ovarian Syndrome (PCOS), Premature Ovarian Insufficiency (POI), endometriosis]
9. Neurocognitive decline and executive function in women with ADHD across the menopausal transition.

Finally, we collaboratively formulated research priorities to improve the debilitating symptoms and quality of life of girls and women with ADHD across the lifespan. While much of the existing research focuses on cisgender women, it is important to acknowledge that ADHD affects a diverse range of individuals, including children, non-binary individuals, and transgender men.

### 2.1 The unique harms of late or incorrect diagnosis

ADHD is not only diagnosed less frequently in girls than boys, but also at a later age (3–5). Often, women with ADHD seek help for other mental health difficulties such as anxiety or depression, rather than ADHD, leading to delayed or missed ADHD diagnoses (4, 6, 7). Compared to male individuals, females with ADHD face higher risks of co-occurring neurodevelopmental and psychiatric conditions, use of psychiatric medications and healthcare services (5, 8, 9). Many risks are worsened with late or missed diagnosis, including teenage pregnancy, risky sexual behaviour, self-harm or eating disorders (10). Late diagnoses also adversely impact relationships, mental health, confidence, and self-esteem in women (11).

Several factors result in delayed diagnosis, including diagnostic practices (e.g., male-biased criteria that may miss female manifestations) and sociocultural reasons (e.g., gendered expectations and masking symptoms (12), and access to inadequate services (13). Women with ADHD often adhere strongly to social norms, using compensatory strategies to mask their symptoms. While these mechanisms help them cope temporarily, they can lead to missed diagnoses, accumulation of secondary comorbid symptoms, and diminished self-esteem (14).

A formal ADHD diagnosis is essential for accessing self-education and other support (e.g., educational or workplace) and treatment (e.g., stimulant medication), which significantly improve long-term outcomes (15–17). However, girls and women are less likely to receive ADHD medications even when diagnosed (3, 18, 19).

#### 2.1.1 Research gaps

Future research should investigate why girls and women are still underdiagnosed or diagnosed late and explore strategies and what symptoms of female ADHD should prompt proper diagnostic measures and improve early identification. Mixed-methods longitudinal studies and the involvement of women with lived ADHD experiences in research are essential to address these gaps.

### 2.2 The role and impact of hormones in female ADHD

For several decades, clinicians have observed changes in the severity of ADHD and mood symptoms during periods of hormonal fluctuations in women with ADHD (20–22). Currently, research is slowly catching up, providing the numbers to back up this clinical observation: the first self-report study of 209 women with clinically diagnosed ADHD found significantly higher premenstrual depressive symptoms than in the general population (45% vs. 28%) (23). Also, PPD symptoms were three times more prevalent (58% vs. 19%), and perimenopausal complaints showed a large effect size (Cohen's d 3.71). Vice versa, ADHD prevalence also has been shown to be increased in women with PMDD (24).

The question remains: why do these symptoms increase during periods of hormonal change in women with ADHD? One proposed mechanism involves the interplay between the neurotransmitter dopamine (25, 26), and fluctuations in oestrogen and progesterone levels throughout the female lifespan (27–32). Both the female sex hormones and dopamine have been associated with cognitive function and they reinforce each other's actions: oestrogen modulates dopamine (33–35), its synthesis, maintenance and the inhibition of its degradation (36, 37). Research in neurotypical women (*n* = 32) found increased hyperactivity and impulsivity during the early follicular and luteal phases (38), especially in those with high trait impulsivity, suggesting that hormonal changes may exacerbate ADHD symptoms especially in those with higher overall levels of ADHD characteristics. Another possible explanation may be inflammatory mechanisms, involved in the complex pathophysiology of ADHD (39–41), and in the menstrual cycle (42–44), however findings are preliminary.

It is understandable that when oestrogen is low or declining in an individual in whom important neurotransmitters such as dopamine are *already* low or dysregulated, these 'shortages' reinforce each other. Thus, women with ADHD may experience increased impairment in their mood, cognition, memory, sleep, and other domains of functioning.

Hormonal decline during perimenopause also increases the risk of cardiovascular disease (CVD), with CVD being the number one cause of death in women in general (45). One study showed that during perimenopause women with lifetime ADHD symptoms were overrepresented in a cardiology clinic (35% of *n* = 300 women) and were two years younger than the other women (46). It is possible that beyond the classical lifestyle risk factors such as a high Body Mass Index (BMI), diabetes, smoking, increased blood pressure and triglycerides, and a sedentary lifestyle, inflammation and stress-induced microvascular coronary artery spasm may play a role in this increased prevalence of CVD in women with ADHD symptoms (47, 48).

#### 2.2.1 Research gaps

Understanding the interplay between female hormones and neurotransmitters is crucial for improving the diagnosis and treatment of ADHD in women. Given the impact of hormonal fluctuations on mood, cognition, and overall functioning, individualised treatment strategies—such as optimising stimulant dosages across menstrual phases (49), and/or offering SSRIs (50) and/or hormonal therapy (51), may improve symptom management. Future research should identify the mechanisms underlying these interactions as well as the interplay with cardiovascular health, and develop potential therapeutic targets to mitigate the heightened symptom burden.

### 2.3 Executive function challenges among girls and women with ADHD

Deficits in executive functions (EF) — such as inhibition, planning, working memory and task-switching — are a hallmark of ADHD. However, several studies identify sex differences in their presentation (52, 53). For instance, boys and men show worse response inhibition, slower processing speed, and more deficits in motor functioning (54–59) than girls and women. A recent meta-analysis confirmed greater deficits in inhibition and in cognitive flexibility in boys and men compared to girls and women, but no differences in working memory, planning and attention (60). In other studies, female children and adults with ADHD may experience more challenges with vocabulary, intellectual abilities, visuo-spatial reasoning and arousal/speed (55, 56, 59, 61). Girls are also more sensitive to small(er) immediate rewards than boys (62). It is also worth noting that EF may be affected by hormonal changes related to the menstrual cycle. Low oestrogen levels may induce hyperactivity-impulsivity symptoms post-ovulation and inattention symptoms peri-menstrually (27). However, these results must be interpreted with caution considering that a study in children aged 7 to 13 noted no significant sex difference in EF (63), suggesting that differences may emerge later, potentially influenced by pubertal hormonal changes.

Systematic reviews of magnetic resonance imaging studies have elucidated anatomical and functional alterations underlying executive dysfunction in ADHD, involving fronto-striato-parieto-cerebellar networks (64, 65). Most studies focused on male or mixed samples, however, there is preliminary evidence of both shared and specific sex-related brain alterations. For instance, studies reported sex-related effects on grey and white matter anatomy in frontal regions subserving impulse control and attention, such as smaller grey matter volumes in girls and white matter volumes in boys, as well as in their functional interaction with the default mode network during attention-demanding tasks, which was more compromised in boys (66–68). Despite these insights, research on EF in girls and women with ADHD remains limited, as most studies have been male-dominated.

#### 2.3.1 Research gaps

Larger and more diverse female ADHD samples are essential to understand better sex-specific neuropsychological and neurobiological patterns. Hormonal interactions (oestrogen and progesterone) and their impact on ADHD symptoms and EF in girls and women require a systematic investigation. Further, longitudinal studies may clarify whether early EF deficits differentially impact longer-term outcomes, guiding preventive, and therapeutic interventions. Future studies may need to take hormonal status into consideration and should be timed according to menstrual phase or take ovulation inhibition into consideration. As prior studies have suggested that functional brain differences at various time points in the menstrual cycle, future studies need to investigate if intra-individual variability across the menstrual cycle phase is more pronounced than inter-individual variations related to underlying conditions.

### 2.4 Pubertal development in girls with ADHD

Adolescence is a period of social, psychological, and biological change that includes puberty, a time of hormonal changes that lead to reproductive maturity (69). Deviation from standard age of pubertal onset (early or late puberty), is associated with higher risk for later physical (70), mental (71–73) and social problems (74). While there is evidence for deviating pubertal timing in other neurodivergent groups such as autism [e.g., (75, 76)], there is currently limited evidence that ADHD itself is associated with a differential pubertal timing (77–79).

One study found that prepubertal use of stimulants was associated with a later age at menarche (i.e., first menstruation) in an all-female adolescent sample with ADHD. This effect might be driven by a lower BMI in those who started stimulant treatment at prepubertal age (77). Another study in Taiwan found girls with ADHD had higher risk for precocious puberty than girls without, but that stimulant medication had no effect on this association (and overall the condition was very rare, approximately 1 in 24,000 children) (80, 81).

Regarding stimulant use during adolescence, a pharmacovigilance analysis of United States Food and Drug Administration (FDA) report found that female adolescents over the age of 13 using methylphenidate, atomoxetine, and amphetamines, reported different and more frequent adverse events than male individuals (82). We currently do not know why these differences arise. The adolescent period could be associated with differential treatment needs during pubertal development, also since a decrease in hyperactivity (but not inattention) is seen in female adolescents during this period (83). Importantly, adolescence is not merely a period of biological change, but also of rapid social and psychological transition, characterised by shifting societal expectations (27) and greater need for autonomy (84). Both biological and psychosocial change should be taken into account when considering diagnosis and treatment, however *how* this should be done is currently not known.

#### 2.4.1 Research gaps

Research into ADHD and puberty, and into the effects of stimulant use on pubertal development in girls with ADHD is scarce, as is research on the impact of pubertal development on the effectiveness of stimulants. Large longitudinal case-control studies incorporating validated measures, as well as thorough mapping of stimulant treatment history is needed. We currently have little information on pharmacokinetics under the influence of hormonal fluctuations. Here we need both preclinical work, as well as pharmacological MRI in women and men with and without ADHD. Moreover, we know little about differential treatment needs in the light of changing ADHD characteristics and (societal) expectations, which could be investigated using mixed methods designs to elucidate ADHD specific expectations arising during puberty.

### 2.5 Sexual health and risky sexual behaviour

Women with ADHD are more likely to engage in risky sexual behaviours compared to their non-ADHD peers. These include increased likelihood of having multiple sexual partners and choosing risky sexual partners (85), earlier sexual activity (86), less frequent use of contraception (87), higher frequency of sexually transmitted diseases (88), and higher rates of unplanned and teenage pregnancies (89, 90).

Regarding the quality of their sexual life, women with ADHD also present with less sexual satisfaction and more sexual dysfunctions (91). These behaviours are often linked to emotional dysregulation, impulsivity, and oppositional symptoms (92). Women might feel strong expectations to fit in socially and feel under more peer pressure to act a certain way (11, 93). Additional social factors such as use of alcohol or drugs and staying out all night have been linked to increased risks of sexual risk-taking behaviours (94).

Finally, it is important to note that women with ADHD are also more likely than their peers to be victims of sexual abuse (95) and sexual victimisation (96, 97). Such risk increases in the event of identification with gender and sexual minority groups; women with ADHD are in fact more prone to identify as transgender and/or non-heterosexual gender identity compared to neurotypical individuals (92, 98, 99).

#### 2.5.1 Research gaps

In addition to these findings, there is a need for more nuanced research focusing specifically on girls and women with ADHD. Future studies should aim to disentangle the roles of socio-demographic correlates and co-occurring conditions (e.g., autism, substance use) and environmental factors (e.g., childhood adversities) in contributing to risky sexual behaviours. Additionally, exploring the effectiveness of non-pharmacological and pharmacological interventions in reducing risky sexual behaviours and their consequences among women with ADHD is crucial.

To address these research priorities, it is essential to conduct large-scale, longitudinal studies that include diverse populations. Raising awareness of these increased risks in clinical settings is also essential for healthcare staff supporting girls and women with ADHD, ensuring a holistic view to ADHD that encompasses their sexual health and safety.

### 2.6 The benefits and risks of hormonal birth control for women with ADHD

Hormonal contraception for women with ADHD is a critical consideration, given their increased risk of early motherhood and engagement in risky sexual behaviour. Women with ADHD are six times more likely to become mothers before age 20, highlighting the importance of effective contraception for this population (89).

Long-acting reversible contraceptives (LARCs: hormonal IUDs, non-hormonal copper IUDs, hormonal implants) show promise for women with ADHD, with 74.2% of young women continuing use beyond 24 months, compared to only 14.9% for short-acting methods (100). This suggests that LARCs may be more suitable contraceptives, given the impulsivity and forgetfulness associated with ADHD.

Combined oral contraceptives (COCs) and progestin-only pills (POPs) have been shown to increase the risk of depression up to five times in women with ADHD compared to unaffected women, regardless of hormonal content (101). Thus, women with ADHD may be more sensitive to adverse mood effects from oral hormonal contraception, and/or mood changes due to forgetting the pill.

#### 2.6.1 Research gaps

Further research is needed to understand the specific interactions between ADHD symptoms, hormonal contraceptives, and mood regulation. Priorities include investigating tailored contraceptive counselling that increase the use of safe and tolerable contraceptive methods in young women with ADHD. Also investigating the effects of oral and non-oral hormonal methods on mood and ADHD symptoms and quality of life (23).

To address these research priorities, longitudinal studies comparing different contraceptive methods among women with ADHD are necessary. Collaboration between psychiatrists, gynaecologists, and endocrinologists is crucial to design comprehensive studies that address both reproductive health and mental health aspects of contraceptive use in women with ADHD.

### 2.7 Maternal ADHD in the peripartum period

Currently, there is scarce evidence about maternal ADHD during pregnancy and after childbirth (the peripartum period). As mentioned earlier, women with ADHD are more likely to experience unplanned pregnancies. They are also more likely to continue smoking and alcohol consumption during pregnancy (102).

Pregnant women with ADHD often experience higher stress levels, less social support, and are at increased risk for depression and anxiety (103). There is a higher incidence of pregnancy and birth complications among mothers with ADHD, including pre-eclampsia, unplanned caesarean sections, premature births, and infections (104). High blood pressure is a symptom of pre-eclampsia, again hinting at a higher risk of cardiovascular diseases in women with ADHD.

Mothers with ADHD have a significantly higher risk (5–6 times) of developing postpartum depression and anxiety disorders (103). Children of mothers with peripartum depression have an increased risk of ADHD symptoms (105). It has been suggested that at least in subgroups of women with peripartum depression, inflammatory mechanisms may play a role (106). The relevance of this for women with ADHD remains to be investigated.

A Canadian study, analysing nearly 900,000 pregnancies in Ontario, found an almost 11-fold increase in ADHD stimulant medication use during pregnancy between 2000 and 2021, with a high rate of discontinuation during pregnancy and a partial resumption postpartum (107). The study also identified specific maternal characteristics associated with stimulant medication use during pregnancy, including lower income, higher BMI, smoking during pregnancy, and concurrent use of other psychotropic medications. Moreover, a study using Swedish registry data, found no increased risk of neurodevelopmental disorders, including ADHD and autism spectrum disorder, in children exposed to ADHD medications *in utero*, consistent across various medication types and exposure durations. The findings, also supported by a meta-analysis combining data of a previous Danish study, provide crucial safety data for methylphenidate, amphetamines, and atomoxetine (108). Other studies, found that the use of methylphenidate during pregnancy and breastfeeding is considered relatively safe, while amphetamine and lisdexamphetamine are contraindicated during breastfeeding due to potential accumulation in the child (102). There are few data about the safety of atomoxetine during breastfeeding, but if a non-stimulant drug is needed, bupropion is emerging as the preferred treatment option (109, 110). ADHD in mothers can affect parent-child interactions, leading to both positive (increased warmth) and negative (increased stress, lax or over reactive parenting) outcomes (102).

#### 2.7.1 Research gaps

There is a need for more research on pregnancy and birth complications, peripartum anxiety and depression in women with ADHD, as well as safe and novel pharmacological and psychological treatment options. Additionally, more studies are required to investigate mother-child bonding and interactions during the early postnatal period. Given that ADHD is highly heritable, with environmental and developmental risk factors also contributing, early infancy represents a particularly vulnerable period. Therefore, research is needed to identify potential targets for prevention and early intervention to support optimal child development and mitigate the risk of adverse outcomes associated with perinatal and early infancy risk factors in children with a genetic predisposition to ADHD.

### 2.8 Hormonal and gynaecological disorders associated with ADHD

Research about hormonal and gynaecological disorders in ADHD remains scarce, despite emerging evidence suggesting that women with ADHD may experience elevated rates of polycystic ovarian syndrome (PCOS) (111–113) and possibly premature (or primary) ovarian insufficiency (POI) (114). Women with endometriosis have an increased risk of a later diagnosis of ADHD (115).

Five to eight percent of women of reproductive age are affected by PCOS, a heterogeneous endocrine syndrome characterised by two or more of the following criteria: hyperandrogenism, anovulation marked by infrequent or absent periods, and polycystic ovaries (116, 117). Prenatal exposure to excess androgens *in utero* may be linked to a higher incidence (42% chance) of ADHD in the offspring of mothers with PCOS (118), notably for female offspring (119), although causality is not established. The putative causal mechanisms linking PCOS and ADHD remain understudied.

In endometriosis, tissue resembling the endometrium is located outside the uterine cavity that causes an inflammatory reaction and sometimes scar tissue [see (120) for clinical guidelines]. Endometriosis occurs in approximately 2%–10% of women, usually during the reproductive years, and is associated with a higher risk of infertility (120). Symptoms can include pain during menstruation, during ovulation or sexual intercourse (dyspareunia), chronic pelvic pain, and fatigue. Women with endometriosis are more likely to be diagnosed with ADHD compared to those without endometriosis and to the general female population in Sweden (115). However, it is not known whether this is genetic confounding, or due to other links between ADHD and endometriosis.

Finally, POI refers to premature menopause (before the age of 40) and occurs in about 1% of women. POI is associated with oestrogen loss, and if untreated, it increases risk for cardiovascular disease, osteoporosis, and dementia (121). The causes of POI are often unknown and can include autoimmune disease or a mutation in the FMR1 gene (fragile X) (114). Women with a premutation of the FMR1 gene and a high number of CGG repeats may be more likely to have ADHD (114), but more studies are needed to investigate links between POI and ADHD.

#### 2.8.1 Research gaps

Amid calls to study the impact of reproductive life transitions and the menstrual cycle on female ADHD (13, 122, 123), substantial research is needed to bridge the gaps in the understanding of hormonal and gynaecological disorders in female ADHD. Too little is known about whether women with ADHD are at higher risk of these disorders than their counterparts, as well as whether hormonal and gynaecological disorders play a role in inter-individual differences between women with ADHD in terms of ADHD presentation, mental health, and treatment outcomes. Furthermore, there may be complex underlying relationships which remain to be explored between ADHD, hormonal and gynaecological disorders, and inflammatory processes. Indeed, hormonal and gynaecological disorders have been linked to inflammatory processes (124–129) as well as cardiometabolic risk factors (130), and preliminary studies – if heterogeneous and sometimes contradictory – provide possible evidence of elevated inflammatory markers in ADHD (40, 131).

### 2.9 Neurocognitive decline and executive function in women with ADHD across the menopause transition

Women with ADHD experiencing menopause face unique cognitive challenges. There is evidence for neurocognitive and executive function decline in this group as post-menopausal women frequently report subjective cognitive decline (SCD) associated with reductions in medial temporal lobe volume, attention, verbal and working memory (132–134). This is also described in women taking oestrogen-decreasing treatments (134). SCD may be a marker for future Alzheimer's disease (134).

While less is known about neurocognition in women with ADHD, a genome wide association study showed an association with earlier onset of natural menopause (135), which is linked to Alzheimer's disease and late-life memory decline (136, 137). In older adults, ADHD increased susceptibility to cognitive impairment, especially with white matter hyperintensities (138). Women with ADHD have increased risk for (peri)menopausal symptoms (23), and a study assessing women with and without ADHD found that sleep problems, anxiety, and depression accounted for poorer executive functioning among post vs. premenopausal women (139).

No studies have specifically examined executive function decline in women with ADHD across the menopausal transition, but overlaps can be inferred from those without ADHD. Menopausal symptoms, like difficulty concentrating, can resemble ADHD, suggesting that hormonal changes may contribute to ADHD symptoms during midlife (140). Small studies found that treatments for ADHD (lisdexamphetamine and atomoxetine) significantly improved executive function difficulties in peri- and postmenopausal women without an ADHD diagnosis (141, 142). Menopausal sleep disorders impact cognitive function and overlap with ADHD symptoms (29, 30). The prevalence of sleep disorders among postmenopausal women is 51.6% (143) and about 60% in adults with ADHD (similar in men and women (144),. Given that (earlier) menopause, sleep loss and ADHD all contribute to poorer executive functioning, it is plausible that post-menopausal women with ADHD experience compounded executive function decline.

#### 2.9.1 Research gaps

Longitudinal analyses of cognition and brain imaging studies among women with and without ADHD are important across the stages of menopause. Additionally, the impact of menopausal hormone therapy on cognition and the effectiveness of ADHD treatments should be investigated.

## 3 Concluding discussion

ADHD in girls and women has long been under-recognised, under-researched, and under-treated, and research was limited to cisgender women. This position paper identifies multiple critical knowledge gaps and highlights the need for a paradigm shift that considers more the complex interplay between neurodevelopment, hormonal dynamics, and gender-specific psychosocial factors.

• Longitudinal inclusive cohort studies that track individuals with ADHD of different genders across life stages to examine developmental trajectories, executive function profiles, and mental and physical health outcomes.
• Clinical trials exploring sex and gender-specific pharmacological treatments, including menstrual-cycle-adjusted stimulant dosing, SSRI treatment, and hormonal interventions (e.g., contraceptives, menopausal hormone therapy) to optimise ADHD symptom management.
• Comorbidity-focused research examining the bidirectional associations between ADHD and conditions such as PMDD, PCOS, endometriosis, cardiovascular disease, and cognitive decline, with attention to inflammatory pathways.
• Studies on the impact of puberty, pregnancy, menopause, and transition on cognitive function, risk-taking behaviours, and treatment efficacy, particularly in relation to stimulant pharmacokinetics and hormonal fluctuations.
• Research on the peripartum period, including risks of postpartum depression and impaired mother-infant bonding, to inform early interventions and impact of intergenerational ADHD transmission.
• Diagnostic innovation, including the development of sex and gender-sensitive tools that account for female-typical symptom expression, masking behaviours, and internalising comorbidities.

Addressing these research gaps requires interdisciplinary collaboration across psychiatry, endocrinology, gynaecology, cardiology, and neuroscience. By prioritising hormonal and inflammatory pathways and adopting inclusive, lifespan-focused research designs, the field can advance towards more effective, gender-sensitive ADHD care.
